# Potential Therapeutic Significance of Laminin in Head and Neck Squamous Carcinomas

**DOI:** 10.3390/cancers13081890

**Published:** 2021-04-15

**Authors:** Nathalia Meireles Da Costa, Fábio A. Mendes, Bruno Pontes, Luiz Eurico Nasciutti, Luis Felipe Ribeiro Pinto, Antonio Palumbo Júnior

**Affiliations:** 1 Programa de Carcinogênese Molecular, Instituto Nacional de Câncer—INCA, Rua André Cavalcanti, 37-Centro, Rio de Janeiro 20231-050, Brazil; nathalia.meireles@inca.gov.br (N.M.D.C.); lfrpinto@inca.gov.br (L.F.R.P.); 2 Instituto de Ciências Biomédicas, Prédio de Ciências da Saúde—Cidade Universitária, Universidade Federal do Rio de Janeiro, Ilha do Fundão, A. Carlos Chagas, Rio de Janeiro 21941-902, Brazil; mendes@icb.ufrj.br (F.A.M.); bpontes@icb.ufrj.br (B.P.); 3 Laboratório de Interações Celulares, Instituto de Ciências Biomédicas, Prédio de Ciências da Saúde—Cidade Universitária, Universidade Federal do Rio de Janeiro—UFRJ, Ilha do Fundão, A. Carlos Chagas, 373-bloco F, sala 26, Rio de Janeiro 21941-902, Brazil; luiz.nasciutti@histo.ufrj.br

**Keywords:** head and neck cancer, oral cancer, pharyngeal cancer, laryngeal cancer, extracellular matrix, laminin, Laminin-111, Laminin-332, Laminin γ2, LAMC2

## Abstract

**Simple Summary:**

Head and neck cancers (HNC) account for approximately 500,000 new cases of tumors annually worldwide and are represented by upper aerodigestive tract malignant neoplasms, which particularly arise in oral cavity, larynx, and pharynx tissues. Thus, due to the biological diversity between the upper aerodigestive organs, and to the heterogeneity of risk factors associated with their malignant transformation, HNC behavior, and prognosis seem to strongly vary according to the tumor site. However, despite to the heterogeneity which characterizes head and neck tumors, squamous cell carcinomas (SCC) represent the predominant histopathologic HNC subtype. In this sense, it has been reported that SCC tumor biology is strongly associated with deregulations within the extracellular matrix compartment. Accordingly, it has been shown that laminin plays a remarkable role in the regulation of crucial events associated with head and neck squamous cell carcinomas (HNSCC) progression, which opens the possibility that laminin may represent a convergence point in HNSCC natural history.

**Abstract:**

Head and neck squamous cell carcinomas (HNSCC) are among the most common and lethal tumors worldwide, occurring mostly in oral cavity, pharynx, and larynx tissues. The squamous epithelia homeostasis is supported by the extracellular matrix (ECM), and alterations in this compartment are crucial for cancer development and progression. Laminin is a fundamental component of ECM, where it represents one of the main components of basement membrane (BM), and data supporting its contribution to HNSCC genesis and progression has been vastly explored in oral cavity squamous cell carcinoma. Laminin subtypes 111 (LN-111) and 332 (LN-332) are the main isoforms associated with malignant transformation, contributing to proliferation, adhesion, migration, invasion, and metastasis, due to its involvement in the regulation of several pathways associated with HNSCC carcinogenesis, including the activation of the EGFR/MAPK signaling pathway. Therefore, it draws attention to the possibility that laminin may represent a convergence point in HNSCC natural history, and an attractive potential therapeutic target for these tumors.

## 1. Introduction

Head and neck cancer (HNC) are among the most common and lethal tumors worldwide, affecting mostly men and populations from low- and middle-income countries [1,2]. The great majority of HNC originate from the squamous cells lining the mucosal epithelium in head and neck sites, being collectively named head and neck squamous cell carcinomas (HNSCC). The head and neck anatomical sites more frequently affected by the development of HNSCC are the oral cavity, pharynx, and larynx [3,4]. Together, these three anatomical sites congregate the vast majority of HNSCC cases and the major number of deaths, as previously estimated [1,5] and summarized in Table 1.

The main risk factors associated with these tumors are tobacco smoking and alcohol consumption [6]. Additionally, viral infections caused by Human Papilloma Virus (HPV) and Epstein Barr Virus (EBV) are highly associated with oropharynx and nasopharynx development, respectively [6].

HNSCC presents poor prognosis since most patients are diagnosed presenting advanced-stage tumors [7]. HNSCC treatment protocols can include surgery, radiotherapy, chemotherapy, targeted therapy, or a combination of treatments, depending on the location and stage of the tumor, patient age, and health condition [8]. The high incidence and lethality of these tumors, together with their poor prognosis, draw attention to need for deeper understanding their biology to identify potential biomarkers and therapeutic strategies to improve patient management and survival.

Molecular characterization of HNSCC has shown a great heterogeneity in molecular alterations present in these tumors, not only because of the different sites analyzed together, but also because of the low frequency of specific genetic alterations, even when a specific site is considered [9]. This characteristic hampers the development of target therapies based on the genetic alterations present in the neoplastic cells. However, numerous studies have shown the fundamental role of the extracellular matrix (ECM) during cancer development and progression, as well as its potential as therapeutic targets [10,11,12]. In fact, the relevance of ECM to cancer progression and treatment has long been established and is originated from the observation that an increase in ECM deposition occurs in the evolution of malignant neoplasms and is associated with poor patient prognosis and treatment resistance [13].

ECM includes the interstitial matrix and the basement membrane (BM) [14]. Although the interstitial matrix is composed of polysaccharides and fibrous proteins that fill the spaces between cells and acts as a sort of buffer against mechanical stresses and strains placed on the ECM [14], BM are sheet-like specialized ECM regions that surround most animal tissues [15]. BM functions are quite diverse, not only involving physical roles such as anchoring the epithelium, but also maintaining tissue integrity and acting by storing growth factors and cytokines, functioning as a bridge between physical forces and biochemical signaling [15]. The main components of BM are laminins, collagen IV, nidogens and the proteoglycans perlecan and agrine [15]. Defects in BM assembly or composition result in a multitude of human diseases. Moreover, although traditionally viewed as a protective structure to defend tissues against cancer spread and invasion, BM dysregulation is a hallmark of many cancers [16]. BM degradation by proteases, specifically matrix metalloproteinases (MMP -1, -3, -7, -9, -10, -12, -13 and -19) produced by tumor cells, such as carcinoma, is also followed by the production of their own array of molecules, used as substrates for cancer cell invasion and proliferation. Although tumor-derived BM molecules do not form a similar network as those from normal tissues, there are striking intramolecular interactions followed by dynamic modification occurring in the newly formed BM that are crucial in supporting cancer development [16]. In this sense, laminins are glycoproteins of high molecular weight (approximately 400 to 900 KDa) found in the BM of several epithelial tissues and are presented in the shape of a cross or T, formed by three interlaced chains called α, β and γ. They were first identified in Engelbreth–Holm–Swarm sarcoma, a tumor that produces large amounts of basement membrane [17]. Laminins are known to directly regulate crucial biological events associated with morphogenesis, such as proliferation, adhesion, migration, angiogenesis, and survival. However, alterations in laminin expression pattern as well as activity are associated with pathological events, for instance the carcinogenic process, since they can modulate key cellular processes thus influencing different cellular behaviors [18,19], thus increasing their relevance as a prominent therapeutic target [18]. In addition, several studies have shown that the alterations in laminin expression pattern and activity in tumor tissues are associated with patient outcomes, such as tumor invasiveness and poor prognosis, revealing its potential as prognosis biomarker [20,21].

This review will particularly focus on an important component of the BM, laminin, exploring its role in HNSCC carcinogenesis and offering possibilities for its use as a target therapy for patients with these tumors.

## 2. Laminin and Squamous Cell Carcinomas

In vertebrates there are five types of α chains (α1–5), three types of β chains (β1–3) and three types of γ chains (γ1–3). The three-dimensional arrangement of these chains forms two to three short arms, in their N-terminal region, a small portion of each of the chains, and a single long arm formed by the intertwining of most part of the three chains. The short arms of each chain are formed by the globular domains in addition to the laminin epidermal-growth factor-like domains [22,23,24]. On the other hand, the long arm forms only a single domain, called laminin coiled-coil domain. The C-terminal region in the long arm has five globular domains originating from the α chain, called the laminin globular domain [24,25,26,27]. In total, 18 different laminin isoforms have been found so far and they are named according to the composition of their chains. For example, the best-studied laminin is laminin-111 (LN-111) composed of the α1, β1, and γ1 chains [24].

Laminins can bind to several proteins; however, its four main transmembrane receptors are integrins, dystroglycan, syndecans, and Lutheran blood group glycoprotein. Laminin binding to these receptors is mediated mainly by the laminin globular domains. Integrins are the most studied laminin receptors. They are heteromeric membrane proteins composed of two subunits, α and β. So far, 24 integrins have been identified in mammals and α1β1, α2β1, α3β1, α6β1, α6β4, α7β1, α9β1, and αvβ3 function as laminin receptors (for a more in-depth review, see reference [28]).

Although the literature shows that all laminin isoforms are associated with carcinogenesis, most of the studies indicates that laminin 332 (LN-332) is the one most associated with malignant transformation of squamous tumors [21]. In fact, in normal stratified squamous mucosa, LN-332 expression is associated with epithelial-stromal connection where it exerts a vital role on the maintenance of the cohesion between the BM and these compartments [29].

LN-332 expression has been shown to correlate well with tumor invasiveness [30,31] and poor patient prognosis in different squamous cell carcinomas [31,32]. Overall, tumors overexpressing LN-332 normally arise from tissue sites where LN-332 was physiologically present, although there are some exceptions, such as decreases in LN-332 expression in basal cell carcinomas [33] or in breast and prostate cancers [34,35], tissues known to express LN-332 in physiological situations. Nonetheless, the molecules which compose this “aberrant” BM can interact with LN-332 and, consequently, contribute to squamous cell carcinomas development [21]. In this sense, the carcinogenic role mediated by LN-332 primarily occurs due to its bind to the integrins α3β1 and α6β4, during focal adhesion and anchoring process, respectively [36,37]. However, besides the relevance of adhesion/migration process to tumorigenesis, which are associated with the well-established interaction between LN-332 and integrins α3β1 and α6β4, it seems that the activation of EGFR and PI3K signaling pathways by LN-332 proteolytic fragments also play a central role in squamous cell carcinomas development [38,39]. Nevertheless, γ2 chain, as a single subunit, may also favor the adhesion of epithelial cells, since γ2 chain secretion is necessary for the incorporation of LN-332 into the BM [40]. Furthermore, Ogawa and colleagues demonstrated that the short arm of γ2 chain can induce cell adhesion by preventing the phosphorylation of β4 integrin, through a mechanism that involves the binding of γ2 chain to Syndecan-1 [41]. Ultimately, the key role played by LN-332 in epithelia homeostasis is also observed in squamous tissue malignization, where its functions are not restricted only to the assembly of BM, but also comprises the regulation of signaling pathways associated with adhesion, migration, and growth of malignant cells.

The role of laminin in genesis and progression of the most frequently HNSCC affected sites—oral cavity, pharynx, and larynx—is the focus of the present review and is discussed below.

## 3. Oral Cavity Squamous Cell Carcinomas and Laminin

Oral cavity squamous cell carcinomas (OCSCC) comprise tumors arising from different anatomical sites, such as lips, oral tongue, floor of mouth, buccal mucosa, upper and lower gums, retromolar trigone, and hard palate [42]. The main etiological factors associated with OCSCC development are tobacco smoking and alcohol consumption, and in Asian countries, betel nut and tobacco chewing have also been associated with these tumors [2,43,44]. Tumor clinical stage at diagnosis greatly impacts on patient prognosis and, unfortunately, OCSCC are usually detected at late stages, when metastasis is frequently detected, conferring poor overall survival to patients [42].

In this sense, ECM seems to play an important role in oral cavity carcinogenesis [21], and the importance of laminin in OCSCC natural history has been increasingly acknowledged during recent decades. Initially, alterations in the expression pattern of distinct laminin were observed in the malignant transformation, conferring an aberrant laminin pattern in these tumors [45,46,47]. Indeed, this aberrant pattern of laminins expression was associated with the worst OCSCC patient prognosis, with laminin overexpression being associated with increased tumor size, presence of lymph node invasion, and treatment resistance, among other parameters [48,49,50], indicating that the presence and deposition pattern of LN-332 may be a potential prognosis marker [51,52,53,54].

However, due to the heterotrimeric structure of LN-332, its biological relevance in oral cavity cancer progression seems to be more complex, since it is known that each subunit possesses its own intrinsic activity [40]. To this extent, it has been consistently reported that the aberrant levels of LN-332 γ2 chain are associated with the invasive phenotype of oral cavity tumor cells, since early local invasion [55,56,57]. Furthermore, immunohistochemistry and morphological analysis of oral cavity carcinomas have previously revealed that the LN-332 γ2 chain is actively expressed in tumor-budding areas surrounded by myofibroblasts, indicating that the γ2 chain may act as a mediator of the invasion process through stromal compartment [58]. This is in accordance with the positive association between overexpression of the LN-332 γ2 chain and decreased survival rates of OCSCC patients observed in the study of Gasparoni and colleagues [59]. Furthermore, among a panel of 131 OCSCC prognosis biomarker candidates, the gene that encodes the γ2 chain (LAMC2) was the one that presented the best performance, being associated with the worst prognosis subtypes [60].

In addition to its prognosis potential, the LN-332 γ2 chain may also be useful as an early diagnosis marker for OCSCC, since it has been shown that dysplastic cells from the oral cavity display high levels of γ2 subunit [61]. Additionally, by using large-scale gene expression analysis, Chen and colleagues showed that LAMC2 represents one of the most promising genes to predict the onset of oral cavity squamous tumors [62].

The association between the expression of the LN-332 γ2 chain and the invasive and metastatic processes of OCSCC has been reported not only by translational studies, but also by functional analysis that showed that the expression of the γ2 chain is associated with enhanced migration and invasion processes [18]. Contrarily, Yuen and colleagues observed that the abrogation of γ2 chain expression was able to increase the invasive capacity of OCSCC cell lineages. Nevertheless, the mechanisms underlying this phenomenon was not reported by the authors [63]. In accordance with Jourquin and colleagues, Oku et al. further demonstrated that claudin-1 leads to an increase in the expression of matrix metalloproteinase 2 (MMP-2) and membrane type 1 matrix metalloproteinase (MT1-MMP), as well to the cleavage rate of laminin γ2 chain by these proteases, inducing the invasive phenotype of OCSCC cells [64]. Therefore, the role of the γ2 chain during the migration of malignant oral cells seems to result from its different assembly modes: present in BM as part of LN-332 structure or as a single chain [65]. In this way, it is known that LN-332 binding to distinct integrin complexes, such as α2β1, α3β1, and α6β4 during the anchoring of epithelial cells represents a fundamental step in cell migration [66]. For instance, the binding of LN-332 to α3β1 integrin is a central event in cell–cell adhesion mediated by e-cadherin, which culminates in the decrease of epithelial cells motility [67]. This is in accordance with the data produced by Yuen and colleagues showing that the abrogation of γ2 chain expression could weak cell–cell adhesion and, in turn, favor cell motility [63]. In addition, cell–cell adhesion also represents a crucial regulator of γ2 chain activity, since the destabilization of tight junction, because of claudin-1 silencing, can decrease the expression of metalloproteinases, such as MT1-MMP and MMP-2, leading to the decrease of the cleavage and activation of γ2 chain [64]. Furthermore, the decrease of cell–cell adhesion due to the down-regulation of e-cadherin expression during the epithelial–mesenchymal transition (EMT) also induces the up-regulation of the γ2 chain and increases cellular migration [68]. Moreover, it is already known that besides representing a mandatory step during EMT, the activation of the transcription factor Snail is also able to down regulate the expression of claudin-1 [69]. Thus, the adhesion weakness promoted by the activation of Snail/EMT program seems to be a central pathway involved in the regulation and activation of the γ2 chain, since this pathway could play a dual regulation of γ2 chain expression/activation. This is because low cadherin expression associated with the EMT program could induce overexpression of the γ2 chain, while Snail activation during the EMT program may down regulate claudin-1 expression, which, in turn, culminates with a decrease in γ2 chain activation.

Moreover, the γ2 chain binding to EGFR may also represent a critical event during tumor progression [12,70]. In this sense, it was already reported that the release of a fragment called DIII after the degradation of the γ2 chain by MMP2 and MT1-MMP can bind to EGFR, culminating not only in a positive regulation of cell motility, but also in the activation of the MAPK pathway [38]. These data sound interesting since the overexpression of the γ2 chain before the invasion of OCSCC tumors [55,56,57] is also associated with the proliferation of oral malignant cells due to the triggering of the MAPK pathway by the γ2 chain DIII fragment. In addition to confirming the relationship between EGFR/MAPK signaling pathways and γ2 chain expression, an elegant study produced by Degen and colleagues demonstrated that the overexpression of the γ2 chain itself may force the activation of EGFR/MAPK signaling [70]. Moreover, functional analysis involving the inhibition of EGFR, by using siRNA approach or its chemical blockage, reinforced the association between EGF pathway and γ2 chain in tumor progression [20]. Therefore, since it was previously reported that amplification of EGFR gene is associated with the overexpression of the γ2 chain in OCSCC [71], it is reasonable to think that a loop mechanism may be involved in the aberrant expression of the γ2 chain in OCSCC development. In this sense, the amplification of EGFR gene may induce the expression of the γ2 chain that, in turn, would hyperactivate the EGFR/MAPK signaling pathway, besides inducing migration and growth of malignant cells, also in a feedback circuit, by increasing γ2 chain expression.

Lately, several studies have shown that the biology of HNSCC could be strongly influenced by miRNAs [72]. In this sense, after identifying that the down-regulation of miR-29s family represents a molecular signature associated with HNSCC, Kinoshita and colleagues showed that γ2 chain is a target of miR-29s. The induction of miR-29s can repress γ2 chain expression, reverting the invasive phenotype of HNSCC cell lines [73]. Furthermore, in a similar way, upon ectopic expression of miR-134 in OCSCC cell lines, it was observed that the down-regulation of LAMC2 culminates in the inactivation of PI3K/AKT signaling [74], which seems to reinforce the involvement of γ2 chain in the activation of MAPK pathway. Furthermore, it was reported that the long non-coding RNA LINC00511 may act as a decoy to the miR-765 in the tongue squamous cell carcinoma (TSCC), avoiding the negative regulation of LAMC2 expression by miR-765 as well the malignant progression of TSCC cells [75]. Finally, it seems that the influence spectrum of γ2 chain in oral squamous tumor development is not limited to the regulation of malignant cell behavior per se, since the release of extracellular vesicle enriched in the γ2 chain by OCSCC cells is able to stimulate the lymphatic endothelial cells to promote the lymphangiogenesis, which represents an important route during metastasis establishment [76].

**Figure 1:**
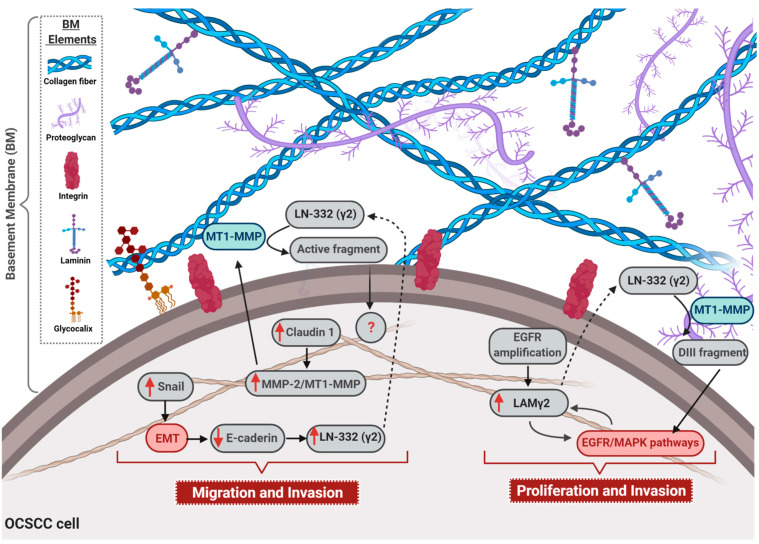
Role of laminin in oral cavity squamous cell carcinomas (OCSCC) progression. The figure illustrates the main mechanisms through which laminin is involved in OCSCC malignant phenotype acquisition. Up-regulation of the transcription factor Snail activates epithelial–mesenchymal transition (EMT), and consequently e-cadherin down-regulation, which enhances laminin 332 (LN-332) γ2 chain expression. Additionally, overexpression of claudin-1 leads to an increase in the expression of matrix metalloproteinase 2 (MMP-2) and membrane type 1 (MT1)—MMP, as well as to the cleavage rate of LN-332 (γ2) by these proteases, culminating in the migration and invasion of OCSCC. Also, EGFR gene amplification is associated with overexpression of γ2 chain (LAM γ2) that, in turn, hyperactivates EGFR/MAPK signaling pathway and, in a feedback circuit, increases LAM γ2expression. Moreover, the secreted LN-332 (γ2) is cleaved by MMP2 and MT1-MMP, generating the DIII fragment that can bind to EGFR, ending up in the activation of EGFR/MAPK pathway. Finally, the activation of EGFR/MAPK signaling pathway enhances proliferation and invasion rates of OCSCC cells. Dotted arrows: molecules secreted by OCSCC cells; solid arrows: activation of cellular events or intracellular signaling pathways; red solid arrow: overexpressed molecules; red boxes: activated signaling pathways; blue boxes: cleavage by MT1-MMP. BM = basement membrane.

Even though the main evidence points to the γ2 chain as a major player in OCSCC development, some studies have revealed that α3 and β3 chains may also be involved in the carcinogenesis of the oral cavity [77,78]. Moreover, despite the relevance of LN-332, the identification of LN-111-derived peptides has reinforced the importance of laminin “active fragments” in the biology of oral cavity cancers, since it was shown that the LN-111-derived peptide, AG73, positively modulates the migration and invasion of OCSCC cell lines by triggering an axis which involves syndecan-1 and β1 integrin receptors, as well as the secretion of matrix metalloproteinase protein 9 (MMP-9) [79]. In addition, activation of β1 integrin, Src, and ERK 1/2 signaling pathways by LN-111-derived peptide C16 seems to be essential for the invasion of OCSCC cells, due to the regulation of invadopodia activity [80]. The mechanisms through which laminin is involved in the OCSCC malignant phenotype acquisition are summarized in Figure 1.

Thus, the involvement of laminin in the progression of OCSCC, through the modulation of key signaling pathways and its consequent impact on crucial cellular mechanisms such as EMT, migration, and invasion is quite clear and justifies its envisagement as a potential therapeutic target.

## 4. Pharyngeal Squamous Cell Carcinomas and Laminin

Pharyngeal tumors comprise neoplasms originating from nasopharynx, oropharynx, and hypopharynx. Nasopharyngeal cancers are the most common among them, accounting for approximately 40% of the pharyngeal tumors, whereas oropharyngeal tumors represent around 30% of them, as well as hypopharyngeal cancers [1]. The great majority of oropharyngeal and hypopharyngeal tumors are squamous cell carcinomas whose development is associated with heavy tobacco and alcohol consumption [81,82]. Another important risk factor associated with the development of oropharyngeal squamous cell carcinomas (OPSCC) is the infection caused by HPV. In recent decades, the incidence of OPSCC associated with tobacco and alcohol abuse has declined gradually worldwide, due to anti-tobacco policies, while the incidence of OPSCC associated with HPV infection is increasing [82]. Similarly, nasopharyngeal tumors are mostly represented by squamous cell carcinomas (NPSCC), nevertheless, the main risk factor associated with their development is the infection with EBV [83,84]. Thus, despite the differences in their site of origin and associated risk factors, pharyngeal tumors are mostly squamous cell carcinomas and, thus, associated with aberrant BM deposition and loss of its structural and functional integrity. Alteration in the expression or deposition of laminin has been extensively investigated as markers of loss of integrity of the BM. In NPSCC, as cited previously, most of the tumors are associated with EBV infection [85,86,87], and part of its transforming potential occurs through the expression of LMP1 oncoprotein. This protein induces cell motility and invasion by activating several different signaling pathways and MMPs, among others. In this sense, induced LMP1 infection in a NPSCC cell line triggered LAMC2 overexpression, as well as that of integrin α6 [88], suggesting that the development of NPSCC is linked to laminin presence. Furthermore, the ectopic expression of the viral oncogenes LMP1, LMP2a, and LMP2b in a normal keratinocyte cell line enhanced epithelial thickness and vacuolization. Additionally, upon ectopic expression of LMP1, LMP2a, and LMP2b, LN-332, whose expression is normally restricted to the basal layer of normal keratinocytes, is strongly detected in the suprabasal layers. This phenomenon is also observed for integrin α6, β4, α3, α5, β1 and other cell adhesion molecules [89], suggesting that EBV infection leads to an altered cell adhesion molecule expression in keratinocytes, compatible with nasopharyngeal carcinomas, and that alteration in laminin is tightly involved in this process.

Additionally, epigenetic alterations are also involved in the differential expression of laminin in NPSCC. Sengupta and colleagues reported a significant up-regulation of LN-332 γ1 chain, as well as seven types of collagen, in nasopharyngeal carcinoma, when compared to non-malignant nasopharyngeal epithelia. The mechanism underlying LN-332 γ1 chain induction is the down-regulation of miR-29c, which specifically targets γ1 and different collagens differentially expressed in NPSCC [90].

Alterations in laminin are also present in OPSCC. Ricci and coworkers demonstrated that LN-332 is aberrantly expressed in oropharyngeal carcinomas. Specifically, while in non-tumor oropharyngeal mucosa the expression of LN-111, LN-332 and collagen IV is displayed as linear and continuous in the BM, in OPSCC this pattern shifts to irregular with loss of linear distribution and fragmentation. In addition, integrin distribution pattern is also irregular and diffuse, being this molecule known for binding to LN-332. Importantly, the observed disruption of basal lamina enhances the invasive and metastatic potential of OPSCC [91], suggesting that the differential laminin expression pattern in tumors is associated with patients’ worst prognosis.

Regarding hypopharynx squamous cell carcinoma (HPSCC), Frenette and coworkers analyzed the involvement of laminin in the metastatic process. In this study, laminin expression pattern was assessed in primary HPSCC, in recurrent or metastatic hypopharyngeal carcinomas, and in non-malignant human keratinocytes. The authors did not observe any significant differences in the levels of laminin expression in the different samples evaluated, nevertheless, they observed that non-malignant keratinocytes secreted lower levels of laminin when compared to tumor samples. Furthermore, HPSCC samples secreted and shed most of the laminin produced and this phenomenon was positively associated with the aggressiveness of the tumor [92]. Still in HPSCC, LN-332 expression was evaluated in a set of samples and its presence was detected in over 90% of the HPSCC investigated, being most of them classified as high LN-332 expression. Additionally, LN-332 was predominantly present in the invasive front of the tumor mass and its expression was positively associated with high infiltrative tumors and presence of vascular invasion. Of note, these were poor prognosis parameters, negatively impacting HPSCC patient overall survival. Finally, other tumor markers were evaluated in the same set of samples, such as E-cadherin, β-catenin and IL-6, and none of them presented significant association with patient clinicopathological parameters or prognosis [93].

Therefore, despite the scarce literature, the data produced so far points out to the role of laminin in the development and/or progression of pharyngeal carcinomas, and consequently, the impact on patient prognosis.

## 5. Laryngeal Squamous Cell Carcinoma and Laminin

Laryngeal tumors represent another very common HNC. Approximately 96% of laryngeal cancers are LSCC [94]. It has been reported that the incidence of LSCC is associated with modern lifestyle factors, including smoking and alcohol consumptions [95]. Furthermore, due to inefficient early diagnosis and prognosis methods, recurrence and metastasis remain the principal causes of death from LSCC [96,97]. Therefore, it is of great importance to find effective early diagnostic indicators and to establish more reliable treatment strategies for LSCC. Among such strategies are the studies that investigate changes in BM. Although early observations had considered BM as a protective structure against cancer spread and metastasis [16], it is now accepted that BM role in cancer invasion and tumor development is much more complex [98].

Modifications in BM structure, particularly those involved with its disorganization or dissolution have been correlated with the biological course of tumors [99], including LSCC, where loss of BM constituents, such as collagen VII in general, has been associated with prognostic factors [100,101,102]. However, tumor cells can secrete their own BM molecules, also used as substrates for invasion and proliferation [98]. One of these molecules is LN-332, which has been shown to be highly expressed in several squamous tumors such as cutaneous, oral and LSCC [21,30,31,32,103]. Particularly for LSCC, Hagedorn and coworkers [102] conducted a study using 26 different carcinomas staged between T1 and T4, with half presenting lymph node metastasis and the majority with diffuse infiltration pattern as poorly differentiated carcinoma. The authors observed the presence of LN-332 in the BM zone of all cases evaluated as well as within tumor cells at the tumor invasion front, indicating a disturbed synthesis and non-polarized distribution of LN-332. These observations agree with Kainulainen et al. [50] and Matsui et al. [104] who also described high expression of LN-332 in oral squamous cell carcinoma, at the tumor-stroma-interface. Although no significant correlation between staining of LN-332 and other clinicopathological parameters and/or prognostic significance could be established. Ricci et al. [91] demonstrated that partial or intense fragmentation of the BM zone are followed by greater loss of the linear distribution of LN-332, which could be considered an additional marker of tumor aggressiveness with increasingly poor prognosis, although they also observed that oropharyngeal carcinomas were found to spread and metastasize more rapidly than laryngeal carcinomas.

High levels of LN-332 could promote the proliferation of tumor cells expressing their interacting integrin and/or non-integrin receptors and should also have a greater metastatic capacity, especially when BM fragmentation exists [105]. It has been suggested that an increase in LN-332 expression (particularly the γ2 chain) is associated with tumor invasion. Pyke and collaborators [106] reported similar observations in colon adenocarcinoma, while Nordermar et al. [32] examined 38 different patients with in situ squamous epithelial carcinomas (CIS) of the larynx and observed that 100% of the CIS lesions that progressed into invasive cancer were LN-332 γ2 chain positive, while only 37% that did not progress to invasive carcinoma showed positivity. Thus, the LN-332 positive laryngeal CIS lesion indicates a high risk of the progression to invasive cancer.

Apart from LN-332 γ2 chain overexpression, other studies have associated the 67-kDa laminin receptor (LR), a nonintegrin receptor, with the metastatic phenotype and poor prognosis in a variety of tumors [107], including LSCC [108]. Zhou and coworkers [108] demonstrated that the expression of LR positively and significantly correlated with the extent of differentiation of LSCC, suggesting the role of this receptor in the proliferative process of this tumor. Moreover, LSCC tumors with cervical metastases expressed higher amounts of LR when compared to those without metastatic features, indicating that LR together with LN-332 could play a critical role in the process of tumor invasion and metastasis. Finally, the authors also speculated that blocking LR expression could be useful to inhibit LSCC tumor aggressiveness at early stage and tested this hypothesis in vitro using a cell lineage derived from a previous LSCC. They showed that the monoclonal antibody against LR inhibited the adhesion of cells to laminin substrates and reduced the invasive capability of the cells to matrigels, suggesting that LR may participate not only in cell migration but also in cell adhesion and matrix dissolution, the three steps involved in tumor invasiveness. Indeed, other studies have shown the crucial role of LR. It has been demonstrated that the binding of tumor cells to laminin induce cells to increase production of LR mRNA [109]. Moreover, binding of LR to laminin induced the secretion of BM degrading enzymes, such as type IV collagenase [110]. In addition, it has been found that LR activated proteolytic enzymes and promoted tumor invasion after ECM degradation [111].

Finally, we must emphasize that this research area still needs to be further investigated. There are still some controversies, such as investigations that did not observe correlations between laminin expression and increases in LSCC metastasis to the neck [112]. It is clear that differences in ECM components and composition have a strong impact on LSCC development and progression. Thus, it is necessary to increase investigations to go ahead unraveling the mechanisms involved to help define possible targets for clinical intervention.

## 6. Laminin as a Therapeutic Target for HNSCC—Future Perspectives

The rising understanding of the mechanisms through which laminin enhances proliferation, migration, and invasion of HNSCC cells, thus contributing to cancer progression, indicates that its suppression may be an interesting therapeutic target for these tumors.

In this context, different strategies could be envisaged to generate potential laminin-based anticancer therapy. For instance, antagonists/neutralizing antibodies could be a promising strategy to block the “oncogenic” effects of laminin, as well as the development of recombinant laminins able to elicit cellular adhesion, but not motility. Also, the generation of molecules that promote the degradation of laminin in tumors could be an intriguing therapeutic strategy. Clearly, one of the challenges of such approaches would be to direct the inhibition effects over laminin expression and/or activity specifically to tumor cells, but not to the healthy ones. Yet, considering HNSCC, LN-111 and LN-332 should be the preferential targets, since these laminins seem to be the most involved with their progression. Of note, the use of a LN-332 antagonist antibody was already tested in skin squamous cell carcinomas (SSCC) demonstrating encouraging and anti-tumor specific results [113]. Additionally, the inhibition of LN-332 expression and/or its functions in tumors, by using synthetic peptides or pharmaceutical reagents, has been investigated and indicated promising outcomes [114,115,116].

In addition to direct target laminin, other molecules intrinsically involved in the activation and/or function of laminin in HNSCC could also be the target of a potential anti-laminin therapy. In this sense, the 37 kDa laminin receptor precursor (LRP)/67 kDa high-affinity LR also represents an attractive target. LRP/LR is a transmembrane receptor that presents high affinity to LN-111 [117], which is one of the main laminins involved in head and neck carcinogenesis. LRP/LR is overexpressed in various cancer types [118] and associated with enhanced tumor invasive potential [107]. Additionally, LR plays an important role in LSCC carcinogenesis, as discussed above. Currently, there are patented approaches targeting LRP/LR as anti-tumor therapy, such as monoclonal antibodies and small interfering RNAs that have already demonstrated to be effective in preventing cell adhesion, invasion and survival in different tumor types [119].

MMPs are also crucial for laminins activation and could also be suggested as an interesting target for anti-laminin therapy. In fact, in recent decades, MMPs have been suggested and evaluated as potential therapeutic target for distinct diseases due to their crucial role in ECM remodeling, but without high success rate. The main reason for this is the fact that there are 23 different MMPs in humans, sharing high structural homology and substrate specificity overlap, but without functional redundancy. This means that each MMP is unique concerning the effect caused on ECM properties [120]. Thus, MMPs inhibitors usually affect more than one metalloproteinase, due to their structural homology, generating undesired off-target effects. Nevertheless, in recent years, the knowledge of MMPs activity has greatly improved, enabling the development of the next-generating MMP inhibitors that are highly specific and capable of discriminating between homologous MMPs. The next-generating of MMP inhibitors includes small molecules and antibody-based inhibitors that unveil new potential anti-laminin therapeutic opportunities [121].

However, it seems that laminin may influence the therapeutic response to classical and new treatment approaches, since it has been reported that laminin could play a key role in chemotherapy and immunotherapy treatments. It was already reported that the ratio between laminin 411 and laminin 511 may affect the migration and polarization of leukocytes that, in turn, could disturb the shift from the immunotolerant to immunoreactive state, thus indirectly impacting on immunotherapy response [122]. However, as previously discussed, laminin 332 and its γ2 chain represent the main laminin subtype associated with natural history of squamous tumors from head and neck [21]. In this way, the study performed by Ly and colleagues shed some light on the mechanisms involved in the immune responsiveness of anti-PD-1 therapy, since it clarifies the intimal association between TGF-β1 and LN-332 γ2 expression, in lung and esophageal cancer patients anti-PD-1 therapy escape, due to a complex circuit which involve the triggering of c-Jun N-terminal kinase (JNK) and AP1 [123]. Moreover, these data seem to reinforce the relevance of the tumor microenvironment during the therapeutic management, since they reveal that the immunosuppressive cytokine TGF-β1 released by cancer-associated fibroblasts (CAFs), was able to induce the overexpression of LN-332 γ2 that, in turn, blocked the entering of T cells in the tumor tissue. This culminates with poor response to immunotherapy mediated by PD-1 blockage. Finally, alterations in the expression pattern of laminin after chemotherapeutic approach [124,125], may also reveal its influence on this therapy, otherwise, no mechanism has been already described until the present moment.

Finally, the feedback activation loop between LAMγ2 and EGFR signaling pathway in OCSCC (summarized in Figure 1) points out the opportunity of using EGFR anti-tumor target therapy as, or in combination with, an anti-laminin approach. Of note, one of the few currently available molecular target therapies for HNSCC is the EGFR monoclonal antibody, cetuximab. It works as a radiosensitizer and is employed alone or in combination with chemotherapy. Nevertheless, cetuximab, and other EGFR target drugs, still do not reach high efficiency rate for HNSCC treatment [118,126]. Considering the multimodal therapeutic approach preconized for HNSCC, one could suggest that the addition of another molecular target capable of modulating EGFR/MAPK signaling pathway—laminin-based therapy—could improve their efficacy and, thus, patient management and prognosis.

Taken together, this review summarizes the main data that leads to the proposition that laminin is a feasible and potential therapeutic target to be further investigated and explored for HNSCC patient treatment. Laminin has already been suggested as a key therapeutic target for other tumor types [20,119,127], reinforcing its potential as anti-tumor therapy.

## 7. Conclusions

**Figure 2:**
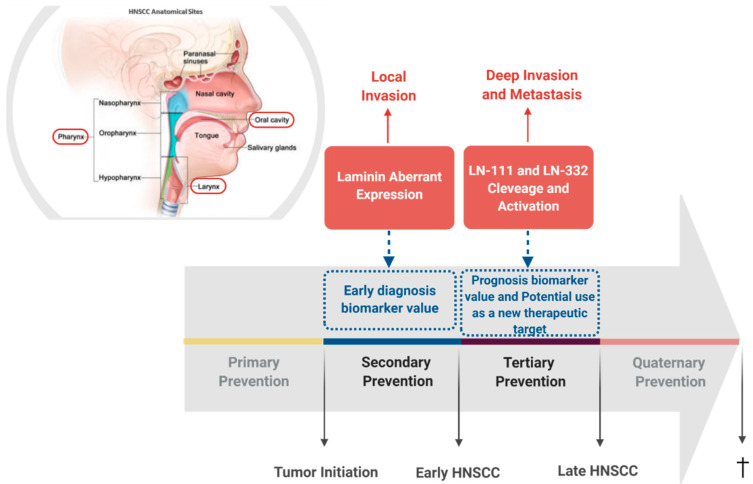
Laminin involvement in head and neck squamous cell carcinomas (HNSCC) progression. Schematic representation of HNSCC most frequent affected anatomical sites and summary of the already identified involvement of laminin in HNSCC, pointing out its intervention potential in tumor’s natural history. Head and neck anatomical sites from where the squamous cell carcinomas discussed in this review arise are highlighted by a red frame. Secondary prevention is highlighted since the aberrant expression pattern of laminin in HNSCC unveils a potential value of its use as early diagnosis biomarker. Likewise, tertiary prevention is also highlighted since cleavage and activation of laminin trigger intracellular signaling pathways that lead to the acquisition of a malignant phenotype and may represent a potential new therapeutic target. Additionally, cleavage and activation of laminin are associated with patient outcome, indicating a potential value of its use as prognosis biomarker.

In this review, we discussed the clear contribution of laminin for the progression of HNSCC, particularly for those of oral cavity, pharynx, and larynx. In this sense, the aberrant expression of LN332 seems to represent the main characteristics which connect the distinct head and neck squamous tumors, since several studies have been shown that the LN332, and particularly its γ2 chain overexpression, are associated with tumor progression of oral cavity, pharynx, and larynx tissues. However, in the development of OCSCC, despite the relevance of LN332/γ2 chain expression, the biological fragments which arise from the degradation not only from LN332 but also from LN111 may play a remarkable role in the carcinogenesis of oral tissue. In fact, it was previously reported that the laminin fragments activate EGFR/MAPK as well as PI3K/AKT signaling pathways, consequently triggering crucial cellular mechanisms, such as EMT, migration, and invasion (Figure 1). Although the scarce literature demonstrates that laminin is involved with the progression of pharyngeal and laryngeal squamous cell carcinoma tumors towards a more aggressive and invasive phenotype, the mechanisms underlying this phenomenon are not yet reported, mainly because of limited in vitro and in vivo models currently available for the study of these tumors. Nevertheless, based on tumor similarities, one could suggest that similar signaling pathways may be modulated in pharyngeal and laryngeal squamous cell carcinomas, representing common mechanisms by which laminin plays a role in HNSCC progression. Therefore, the data discussed here draws attention to the possibility that laminin may represent a convergence point in HNSCC natural history, and an attractive potential therapeutic target for these tumors (Figure 2).

## Figures and Tables

**Table 1:** Estimated numbers of new cases and deaths per year worldwide for the most common types of head and neck squamous cell carcinomas.

Type of Head and Neck Squamous Cell Carcinoma	New Cases per Year Worldwide [1,5]	Deaths per Year Worldwide [1,5]
Oral Cavity Squamous Cell Carcinoma	355,000/male-to-female incidence ratio of 2:1	177,000
Pharyngeal Squamous Cell Carcinoma	302,000	159,000
Laryngeal Squamous Cell Carcinoma	177,000/male-to-female incidence ratio of 7:1	95,000

## Abbreviations

HNC	Head and Neck cancers
SCC	squamous cell carcinomas
HNSCC	Head and neck squamous cell carcinomas
ECM	Extracellular matrix
LN-111	Laminin subtype 111
LN-332	Laminin subtype 332
BM	Basement membrane
EGF	Epidermal-growth factor
EGFR	Epidermal-growth factor
MAPK	Mitogen-activated protein kinase
HPV	Human papiloma virus
EBV	Epstein Barr Virus
PI3K	Phosphoinositide 3-kinase
OCSCC	Oral cavity squamous cell carcinomas
MMP-2	matrix metalloproteinase 2
MT1-MMP	membrane type 1 matrix metalloproteinase
EMT	epithelial–mesenchymal transition
DIII	γ2 chain fragment
TSCC	tongue squamous cell carcinoma
AKT	Protein kinase B
RNA	Ribonucleic acid
MMP-9	matrix metalloproteinase 9
Src	Proto-oncogene tyrosine-protein kinase Src
ERK 1/2	Extracellular signal-regulated kinases 1 and 2
OPSCC	oropharyngeal squamous cell carcinomas
NPSCC	nasopharyngeal squamous cell carcinomas
LMP1, 2a and 2b	Latent membrane proteins 1, 2a and 2b
LAMC2	gene that encodes the γ2 chain
HPSCC	hypopharyngeal squamous cell carcinomas
IL-6	Interleukin 6
LSCC	Laryngeal squamous cell carcinomas
CIS	in situ squamous epithelial carcinomas
LR	Laminin receptor
SSCC	Skin squamous cell carcinomas
LRP	Laminin receptor precursor
